# Supplementary material for: Green and efficient three-component synthesis of 4H-pyran catalysed by CuFe2O4@starch as a magnetically recyclable bionanocatalyst
Source: R Soc Open Sci. 2020 Jul 1;7(7):200385. doi: 10.1098/rsos.200385 (PMC7428224; doi:10.1098/rsos.200385)
Supplement: Prof A. Maleki_Figures NMR_ESM.doc [file rsos200385supp1.docx]

***Supporting Data***

**Green and efficient three-component synthesis of 4H-pyran catalyzed by CuFe_2_O_4_@starch as a magnetically recyclable bionanocatalyst**

*Maryam Kamalzare ^a,b^, Mohammad Bayat ^*,a^ and Ali Maleki ^*,b^*

*^a.^Department of Chemistry, Faculty of Science, Imam Khomeini International University, Qazvin, Iran. E-mail: bayat_mo@yahoo.com, m.bayat@sci.ikiu.ac.ir; Tel: +98 (28)33780040*

*^b.^Catalysts and Organic Synthesis Research Laboratory, Department of Chemistry, Iran University of Science and Technology, Tehran 16846-13114, Iran. E-mail: maleki@iust.ac.ir; Fax: +98 21 73021584; Tel: +98 21 73228313.*

| Content | page |
| --- | --- |
| Spectral data of 4H-Pyran 4b and 4c | S2 |
| Figure S1. ^1^H-NMR spectrum of 4b | S3 |
| Figure S2. ^13^C-NMR spectrum of 4b | S4 |
| Figure S3. ^1^H-NMR spectrum of 4c | S5 |
| Figure S4. ^13^C-NMR spectrum of 4c | S6 |

Spectral data of 2-amino-7,7-dimethyl-4-(4-nitrophenyl)-5-oxo-5,6,7,8-tetrahydro-4H-chromene-3-carbonitrile(**4b**)

^1^H NMR (300 MHz, DMSO-*d6*): *δ* = 0.93 (3H, s, CH_3_), 1.02 (3H, s, CH_3_), 2.06 (2H, dd, CH_2_, *^3^J*=15Hz_)_, 2.22 (2H, dd, CH_2_, *^3^J*=15Hz), 4.34 (1H, s, CH), 7.18 (2H, br s, NH_2_), 7.41 (2H, d, Ar, *^3^J*= 8.1 Hz), 8.14 (2H, d, Ar, *^3^J*= 5.4 Hz). ^13^C NMR (75 MHz, DMSO-*d6*): 27.37 (2CH_3_), 28.78 (C(CH_3_)), 32.07 (CH_2_), 35.95, 50.3 (CH_2_), 57.99 (C(CN)), 112.09, 119.89, 124.30 (CN), 129.30, 146.7, 152.81, 159.16, 163.55, 196.

Spectral data of 2-amino-7,7-dimethyl-4-(2-nitrophenyl)-5-oxo-5,6,7,8-tetrahydro-4H-chromene-3-carbonitrile (**4c**)

^1^H NMR (300 MHz, DMSO-*d6*): *δ* = 0.86 (3H, s, CH_3_), 0.99 (3H, s, CH_3_), 1.96 (2H, dd, CH_2_, *^2^J*=15.9Hz), 2.16 (2H, dd, CH_2_, *^2^J*=15 Hz), 4.91 (1H, s, CH), 7.19 (2H, br s, NH_2_), 7.32 (1H, d, Ar, *^3^J*=7.5 Hz), 7.32-7.67 (1H, m, Ar),7.78 (1H, d, Ar, *^3^J*=8.1Hz). ^13^C NMR (75 MHz, DMSO-*d6*): *δ* = 27.21 (2CH_3_), 28.75 (C(CH_3_)), 30.72(CH), 32.31 (CH_2_), 50.34 (CH_2_), 56.72 (C(CN)), 113.1, 119.48, 124.24 (CN), 128.39, 130.59, 134.02, 139.42, 149.59, 159.74, 163.22, 196.48.

Figure S1. ^1^H-NMR spectrum of **4b**

Figure S2. ^13^C-NMR spectrum of **4b**

Figure S3. ^1^H-NMR spectrum of **4c**

Figure S4. ^13^C-NMR spectrum of **4c**
